# *cis*-Dichloridobis­(5,5′-dimethyl-2,2′-bipyridine)­manganese(II) 2.5-hydrate

**DOI:** 10.1107/S1600536811021805

**Published:** 2011-06-11

**Authors:** Lívia Batista Lopes, Charlane Cimini Corrêa, Renata Diniz

**Affiliations:** a Núcleo de Espectroscopia e Estrutura Molecular (NEEM), Department of Chemistry - Federal University of Juiz de Fora - Minas Gerais, 36036-900, Brazil

## Abstract

The metal site in the title compound [MnCl_2_(C_12_H_12_N_2_)_2_]·2.5H_2_O has a distorted octa­hedral geometry, coordinated by four N atoms of two 5,5′-dimethyl-2,2′-dipyridine ligands and two Cl atoms. Two and a half water molecules of hydration per complex unit are observed in the crystal structure. The compounds extend along the *c* axis with O—H⋯Cl, O—H⋯O, C—H⋯Cl and C—H⋯O hydrogen bonds and π–π inter­actions [centroid-centroid distance = 3.70 (2) Å] contributing substanti­ally to the crystal packing. The Mn and one of the water O atoms, the latter being half-occupied, are located on special positions, in this case a rotation axis of order 2.

## Related literature

For the structures and applications of bipyridine and analogous ligands, see: Hazell (2004 ▶); Bakir *et al.* (1992 ▶); Cordes *et al.* (1982 ▶); Hung-Low *et al.* (2009 ▶). For the structure and applications of 5,5′-dimethyl-2,2′-dipyridine, see: Marandi *et al.* (2009 ▶); van Albada *et al.* (2005 ▶). For weak inter­molecular inter­actions, see: Calhorda (2000 ▶); Desiraju (1996 ▶); Janiak (2000 ▶).
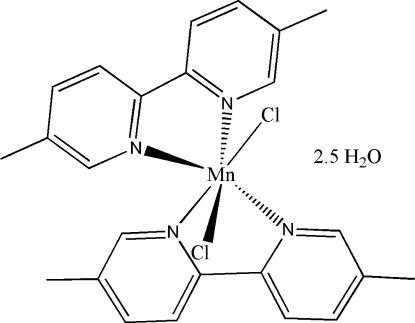


## Experimental

### 

#### Crystal data

[MnCl_2_(C_12_H_12_N_2_)_2_]·2.5H_2_O
*M* *_r_* = 539.35
Monoclinic, 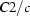

*a* = 18.6703 (9) Å
*b* = 14.0598 (4) Å
*c* = 12.0536 (7) Å
β = 122.430 (7)°
*V* = 2670.6 (2) Å^3^
*Z* = 4
Mo *K*α radiation
μ = 0.72 mm^−1^
*T* = 293 K
0.47 × 0.35 × 0.34 mm

#### Data collection

Oxford Diffraction Xcalibur Atlas Gemini ultra diffractometer
Absorption correction: analytical [*CrysAlis PRO* (Oxford Diffraction, 2008 ▶) based on expressions derived by Clark & Reid (1995 ▶)] *T*_min_ = 0.470, *T*_max_ = 0.697
11860 measured reflections
3317 independent reflections
2499 reflections with *I* > 2σ(*I*)
*R*_int_ = 0.025

#### Refinement

*R*[*F* ^2^ > 2σ(*F* ^2^)] = 0.043
*wR*(*F* ^2^) = 0.150
*S* = 1.09
3317 reflections
155 parameters
H-atom parameters constrained
Δρ_max_ = 0.72 e Å^−3^
Δρ_min_ = −0.31 e Å^−3^

### 

Data collection: *CrysAlis PRO* (Oxford Diffraction, 2008 ▶); cell refinement: *CrysAlis PRO*; data reduction: *CrysAlis PRO*; program(s) used to solve structure: *SHELXS97* (Sheldrick, 2008 ▶); program(s) used to refine structure: *SHELXL97* (Sheldrick, 2008 ▶); molecular graphics: *ORTEP-3 for Windows* (Farrugia, 1997 ▶) and *Mercury* (Macrae *et al.*, 2006 ▶); software used to prepare material for publication: *PLATON* (Spek, 2009 ▶).

## Supplementary Material

Crystal structure: contains datablock(s) global, I. DOI: 10.1107/S1600536811021805/im2285sup1.cif

Structure factors: contains datablock(s) I. DOI: 10.1107/S1600536811021805/im2285Isup2.hkl

Additional supplementary materials:  crystallographic information; 3D view; checkCIF report

## Figures and Tables

**Table 1:** Hydrogen-bond geometry (Å, °)

*D*—H⋯*A*	*D*—H	H⋯*A*	*D*⋯*A*	*D*—H⋯*A*
O1—H1*A*⋯Cl1	0.84	2.42	3.243 (3)	168
O1—H1*B*⋯Cl1^i^	0.81	2.73	3.358 (4)	136
O2—H2*A*⋯O1	0.86	2.16	2.951 (6)	153
C3—H3⋯O1^ii^	0.93	2.49	3.257 (5)	140
C6—H6*A*⋯Cl1^i^	0.96	2.79	3.717 (4)	162

Symmetry codes: (i) 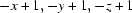; (ii) 
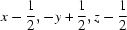
.

**Fig. 1.**
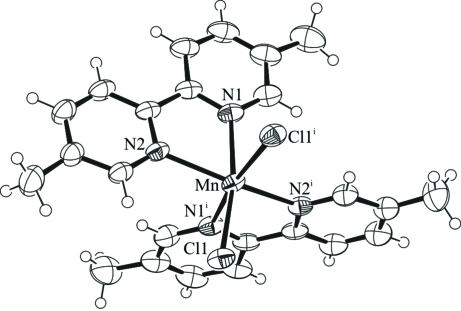
Molecular structure of the complex unit of the title compound [Mn(C12H12N2)2Cl2] × 2.5 H2O. Water molecules were omitted for better visualization. Symmetry code: i (1 - x, y, 1/2 - z).

**Fig. 2.**
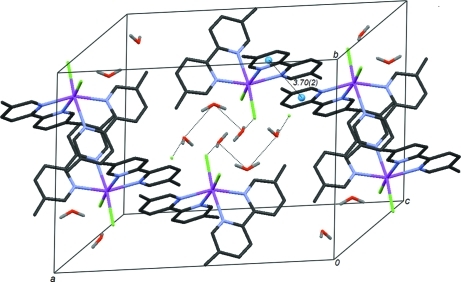
Unit cell of the compound [Mn(C12H12N2)2Cl2] × 2.5 H2O (z = 4), hydrogen bonds chain extending along the c axis and π-π interactions between the rings aromatic ligand dmdpy are depicted as dashed lines.

## supplementary crystallographic information

### Comment

Bipyridine and analogous ligands are commonly used in the formation of different complexes with a general variety of transition metals (Hazell, 2004; Bakir *et al.*, 1992; Cordes *et al.*,1982; Hung-Low *et al.*,2009;). The ligand 5,5'-Dimethyl-2,2'-dipyridine (abbreviated as dmdpy) acts as a chelator and usually gives rise to monomeric compounds (Marandi *et al.*, 2009). Only a limited number of X-ray crystal structures with the ligand dmdpy has been published (van Albada *et al.*, 2005). In this study we used the ligand 5,5'-dimethyl-2,2'-dipyridine and manganese chloride tetrahydrate. This mixture resulted in the compound [Mn(C_12_H_12_N_2_)_2_Cl_2_] × 2.5 H_2_O (Scheme 1).

The molecular structure of the complex unit of the titlecompound [Mn(C_12_H_12_N_2_)_2_Cl_2_] × 2.5 H_2_O is shown in Figure 1. The metal site is coordinated by four nitrogen atoms N1, N2, N1^i^ and N2^i^ of the ligand dmdpy and two chlorides Cl1 and Cl1^i^ adopting a distorted octahedral geometry as evidenced by the Mn—N1 distances (2.3111 (19) Å), Mn—N2 (2.245 (2) Å) and Mn—Cl1 (2.4702 (6) Å). The Mn atom is located in a special position, which in this case is a rotation axis of order 2.

The compound crystallizes in the monoclinic system and its unit cell is shown in Figure 2. The compound [Mn(C_12_H_12_N_2_)_2_Cl_2_] × 2.5 H_2_O is a complex that stretches along the crystallographic *c* axis with the molecular entities being interconnected by weak hydrogen bonds (Desiraju, 1996; Calhorda, 2000) shown in Figure 2. These hydrogen bonds are formed by the interaction between oxygen atoms of water molecules O1 and O2 and the chlorine atom Cl1 which is coordinated to the metal. The distances O1—O2 and O1—Cl1 charge 2.951 (6) Å and 3.243 (3) Å, respectively. π-π interactions between aromatic rings of the nitrogen ligand dmdpy are also shown in Figure 2. These interactions contribute substantially to the crystal packing (Janiak, 2000). In this compound the centroid-centroid distance is 3.70 (2) Å, and there was a substantial overlap between the aromatic rings of the ligand dmdpy, being centroid-plane distance of 3.45 (1)Å and the horizontal displacement of 1.37 (2) Å.

### Experimental

All chemicals were obtained commercially and used without further purification. The complex was synthesized by mixing of 0.38 mmol of dmdpy dissolved in ethanol and 0.38 mmol of MnCl_2_ × 4 H_2_O dissolved in water. The mixture was placed under agitation for 40 h. After a few weeks, yellow single crystals suitable for the analysis of X-ray diffraction were obtained (yield: 39%).

### Refinement

H atoms were positioned geometrically and refined using the riding model approximation with C—H = 0.95 Å, and *U*_iso_(H) was refined in group. H atoms of water molecule were located from electron density map, fixed in these positions and assigned the same isotropic displacement parameters for all H atoms.

### Crystal data

**Table:**

[MnCl_2_(C_12_H_12_N_2_)_2_]·2.5H_2_O	*F*(000) = 1120
*M**_r_* = 539.35	*D*_x_ = 1.341 Mg m^−^^3^
Monoclinic, *C*2/*c*	Mo *K*α radiation, λ = 0.71073 Å
Hall symbol: -C 2yc	Cell parameters from 6304 reflections
*a* = 18.6703 (9) Å	θ = 2.9–29.4°
*b* = 14.0598 (4) Å	µ = 0.72 mm^−^^1^
*c* = 12.0536 (7) Å	*T* = 293 K
β = 122.430 (7)°	Prismatic, yellow
*V* = 2670.6 (2) Å^3^	0.47 × 0.35 × 0.34 mm
*Z* = 4	

### Data collection

**Table:**

Oxford Diffraction Xcalibur Atlas Gemini ultra diffractometer	3317 independent reflections
Radiation source: Enhance (Mo) X-ray Source	2499 reflections with *I* > 2σ(*I*)
graphite	*R*_int_ = 0.025
Detector resolution: 10.4186 pixels mm^-1^	θ_max_ = 29.4°, θ_min_ = 2.9°
ω scans	*h* = −25→23
Absorption correction: analytical [*CrysAlis PRO* (Oxford Diffraction, 2008) based on expressions derived by Clark & Reid (1995)]	*k* = −14→18
*T*_min_ = 0.470, *T*_max_ = 0.697	*l* = −16→16
11860 measured reflections	

### Refinement

**Table:**

Refinement on *F*^2^	Secondary atom site location: difference Fourier map
Least-squares matrix: full	Hydrogen site location: difference Fourier map
*R*[*F*^2^ > 2σ(*F*^2^)] = 0.043	H-atom parameters constrained
*wR*(*F*^2^) = 0.150	*w* = 1/[σ^2^(*F*_o_^2^) + (0.094*P*)^2^ + 0.4813*P*] where *P* = (*F*_o_^2^ + 2*F*_c_^2^)/3
*S* = 1.09	(Δ/σ)_max_ < 0.001
3317 reflections	Δρ_max_ = 0.72 e Å^−^^3^
155 parameters	Δρ_min_ = −0.31 e Å^−^^3^
0 restraints	Extinction correction: *SHELXL*
Primary atom site location: structure-invariant direct methods	Absolute structure: no

### Special details

**Table:**

Geometry. All e.s.d.'s (except the e.s.d. in the dihedral angle between two l.s. planes) are estimated using the full covariance matrix. The cell e.s.d.'s are taken into account individually in the estimation of e.s.d.'s in distances, angles and torsion angles; correlations between e.s.d.'s in cell parameters are only used when they are defined by crystal symmetry. An approximate (isotropic) treatment of cell e.s.d.'s is used for estimating e.s.d.'s involving l.s. planes.
Refinement. Refinement of *F*^2^ against ALL reflections. The weighted *R*-factor *wR* and goodness of fit *S* are based on *F*^2^, conventional *R*-factors *R* are based on *F*, with *F* set to zero for negative *F*^2^. The threshold expression of *F*^2^ > σ(*F*^2^) is used only for calculating *R*-factors(gt) *etc*. and is not relevant to the choice of reflections for refinement. *R*-factors based on *F*^2^ are statistically about twice as large as those based on *F*, and *R*- factors based on ALL data will be even larger.

### Fractional atomic coordinates and isotropic or equivalent isotropic displacement parameters (Å^2^)

**Table:**

	*x*	*y*	*z*	*U*_iso_*/*U*_eq_	Occ. (<1)
Mn	0.5000	0.31073 (3)	0.2500	0.03326 (18)	
Cl1	0.56731 (3)	0.43196 (4)	0.42532 (6)	0.0429 (2)	
N1	0.42155 (12)	0.19415 (12)	0.0968 (2)	0.0391 (4)	
N2	0.38759 (11)	0.27687 (13)	0.26398 (18)	0.0359 (4)	
C1	0.37348 (15)	0.32044 (16)	0.3488 (2)	0.0407 (5)	
H1	0.4120	0.3667	0.4034	0.049*	
C5	0.33239 (14)	0.20969 (15)	0.1835 (2)	0.0368 (5)	
C4	0.26252 (15)	0.18577 (17)	0.1891 (3)	0.0466 (6)	
H4	0.2249	0.1393	0.1334	0.056*	
C2	0.30477 (16)	0.30103 (17)	0.3606 (3)	0.0447 (6)	
C3	0.24897 (16)	0.2312 (2)	0.2779 (3)	0.0523 (7)	
H3	0.2023	0.2150	0.2824	0.063*	
C7	0.35069 (14)	0.16493 (14)	0.0903 (2)	0.0386 (5)	
C8	0.29693 (17)	0.09802 (17)	−0.0022 (2)	0.0511 (6)	
H8	0.2475	0.0794	−0.0072	0.061*	
C11	0.43965 (16)	0.15765 (17)	0.0132 (2)	0.0477 (6)	
H11	0.4884	0.1792	0.0182	0.057*	
C10	0.39085 (19)	0.08913 (18)	−0.0818 (3)	0.0552 (7)	
C9	0.3180 (2)	0.05955 (18)	−0.0865 (3)	0.0580 (7)	
H9	0.2833	0.0135	−0.1470	0.070*	
C6	0.2939 (2)	0.3543 (2)	0.4579 (3)	0.0689 (9)	
H6A	0.3391	0.3995	0.5036	0.103*	
H6B	0.2950	0.3105	0.5199	0.103*	
H6C	0.2405	0.3872	0.4128	0.103*	
C12	0.4172 (2)	0.0531 (2)	−0.1719 (3)	0.0782 (10)	
H12A	0.4692	0.0834	−0.1506	0.117*	
H12B	0.3737	0.0673	−0.2613	0.117*	
H12C	0.4257	−0.0145	−0.1614	0.117*	
O1	0.5956 (2)	0.4141 (2)	0.7145 (3)	0.1311 (14)	
H1A	0.5803	0.4187	0.6356	0.197*	
H1B	0.5699	0.4548	0.7272	0.197*	
O2	0.5000	0.2629 (5)	0.7500	0.112 (3)	0.50
H2A	0.5382	0.2904	0.7422	0.168*	0.50

### Atomic displacement parameters (Å^2^)

**Table:**

	*U*^11^	*U*^22^	*U*^33^	*U*^12^	*U*^13^	*U*^23^
Mn	0.0241 (3)	0.0282 (3)	0.0388 (3)	0.000	0.0111 (2)	0.000
Cl1	0.0341 (3)	0.0394 (3)	0.0431 (3)	−0.0026 (2)	0.0127 (3)	−0.0082 (2)
N1	0.0330 (10)	0.0304 (9)	0.0398 (10)	0.0005 (7)	0.0101 (8)	−0.0025 (7)
N2	0.0286 (9)	0.0321 (9)	0.0364 (9)	−0.0054 (7)	0.0105 (8)	0.0017 (7)
C1	0.0339 (11)	0.0390 (12)	0.0416 (12)	−0.0089 (9)	0.0153 (10)	−0.0011 (9)
C5	0.0307 (11)	0.0281 (10)	0.0350 (11)	−0.0042 (8)	0.0067 (9)	0.0071 (8)
C4	0.0370 (12)	0.0404 (13)	0.0477 (14)	−0.0143 (10)	0.0130 (11)	0.0020 (10)
C2	0.0415 (13)	0.0437 (13)	0.0475 (14)	−0.0073 (10)	0.0230 (11)	0.0053 (10)
C3	0.0403 (13)	0.0560 (16)	0.0563 (15)	−0.0153 (12)	0.0231 (12)	0.0050 (12)
C7	0.0364 (11)	0.0249 (9)	0.0354 (11)	−0.0015 (9)	0.0065 (9)	0.0046 (8)
C8	0.0501 (14)	0.0383 (12)	0.0428 (13)	−0.0147 (11)	0.0102 (12)	−0.0001 (10)
C11	0.0422 (13)	0.0402 (12)	0.0487 (14)	0.0020 (10)	0.0163 (11)	−0.0068 (11)
C10	0.0615 (17)	0.0373 (13)	0.0454 (14)	0.0039 (12)	0.0146 (13)	−0.0061 (10)
C9	0.0666 (18)	0.0354 (13)	0.0454 (15)	−0.0120 (12)	0.0125 (13)	−0.0077 (10)
C6	0.0645 (18)	0.080 (2)	0.079 (2)	−0.0247 (17)	0.0493 (17)	−0.0175 (17)
C12	0.092 (3)	0.068 (2)	0.060 (2)	0.0055 (18)	0.0315 (19)	−0.0192 (15)
O1	0.166 (3)	0.157 (3)	0.110 (2)	0.121 (3)	0.101 (2)	0.068 (2)
O2	0.092 (5)	0.047 (4)	0.135 (7)	0.000	0.020 (5)	0.000

### Geometric parameters (Å, °)

**Table:**

Mn—Cl1	2.4702 (6)	C7—C8	1.391 (3)
Mn—N1	2.3111 (19)	C8—C9	1.382 (5)
Mn—N2	2.245 (2)	C9—C10	1.394 (6)
O1—H1B	0.8100	C10—C12	1.502 (6)
O1—H1A	0.8400	C10—C11	1.397 (4)
O2—H2A	0.8600	C1—H1	0.9300
O2—H2A^i^	0.8600	C3—H3	0.9300
N1—C11	1.326 (4)	C4—H4	0.9300
N1—C7	1.347 (3)	C6—H6A	0.9600
N2—C5	1.350 (2)	C6—H6B	0.9600
N2—C1	1.334 (3)	C6—H6C	0.9600
C1—C2	1.391 (5)	C8—H8	0.9300
C2—C3	1.389 (4)	C9—H9	0.9300
C2—C6	1.494 (5)	C11—H11	0.9300
C3—C4	1.381 (4)	C12—H12B	0.9600
C4—C5	1.383 (4)	C12—H12C	0.9600
C5—C7	1.480 (3)	C12—H12A	0.9600
			
Cl1···C1	3.581 (3)	O1^iv^···C3^ii^	3.257 (5)
Cl1···O1	3.243 (3)	N1···N1^ii^	3.259 (3)
Cl1···Cl1^ii^	3.5759 (9)	N1···N2^ii^	3.233 (3)
Cl1···N1^ii^	3.3695 (18)	N1···N2	2.681 (3)
Cl1···O1^iii^	3.358 (4)	N2···C11^ii^	3.337 (4)
O1···O2	2.951 (6)	C3···O1^v^	3.257 (5)
O1···Cl1^iii^	3.358 (4)		
			
Cl1—Mn—N1	170.62 (6)	N1—C7—C5	116.49 (19)
Cl1—Mn—N2	98.59 (5)	C5—C7—C8	122.4 (3)
Cl1—Mn—Cl1^ii^	92.74 (2)	C7—C8—C9	119.1 (3)
Cl1—Mn—N1^ii^	89.55 (5)	C8—C9—C10	120.7 (3)
Cl1—Mn—N2^ii^	98.24 (5)	C9—C10—C12	124.2 (3)
N1—Mn—N2	72.07 (8)	C11—C10—C12	120.2 (4)
Cl1^ii^—Mn—N1	89.55 (5)	C9—C10—C11	115.6 (3)
N1—Mn—N1^ii^	89.65 (7)	N1—C11—C10	124.8 (3)
N1—Mn—N2^ii^	90.40 (8)	C2—C1—H1	118.00
Cl1^ii^—Mn—N2	98.24 (5)	N2—C1—H1	118.00
N1^ii^—Mn—N2	90.40 (8)	C4—C3—H3	120.00
N2—Mn—N2^ii^	155.51 (7)	C2—C3—H3	120.00
Cl1^ii^—Mn—N1^ii^	170.62 (6)	C5—C4—H4	120.00
Cl1^ii^—Mn—N2^ii^	98.59 (5)	C3—C4—H4	120.00
N1^ii^—Mn—N2^ii^	72.07 (8)	C2—C6—H6C	109.00
H1A—O1—H1B	107.00	C2—C6—H6A	110.00
H2A—O2—H2A^i^	127.00	C2—C6—H6B	110.00
Mn—N1—C11	124.8 (2)	H6B—C6—H6C	109.00
Mn—N1—C7	116.40 (15)	H6A—C6—H6C	109.00
C7—N1—C11	118.7 (2)	H6A—C6—H6B	110.00
C1—N2—C5	118.9 (2)	C9—C8—H8	120.00
Mn—N2—C5	118.63 (17)	C7—C8—H8	120.00
Mn—N2—C1	122.49 (17)	C8—C9—H9	120.00
N2—C1—C2	124.1 (2)	C10—C9—H9	120.00
C3—C2—C6	123.5 (3)	N1—C11—H11	118.00
C1—C2—C3	116.3 (3)	C10—C11—H11	118.00
C1—C2—C6	120.2 (3)	C10—C12—H12B	109.00
C2—C3—C4	120.3 (3)	C10—C12—H12C	109.00
C3—C4—C5	119.6 (3)	H12A—C12—H12C	110.00
N2—C5—C7	116.4 (2)	H12B—C12—H12C	109.00
N2—C5—C4	120.8 (2)	H12A—C12—H12B	110.00
C4—C5—C7	122.8 (2)	C10—C12—H12A	109.00
N1—C7—C8	121.1 (2)		
			
N2—Mn—N1—C7	0.40 (15)	Mn—N2—C1—C2	−179.72 (19)
N2—Mn—N1—C11	177.7 (2)	C5—N2—C1—C2	0.1 (3)
Cl1^ii^—Mn—N1—C7	−98.41 (15)	Mn—N2—C5—C4	179.52 (18)
Cl1^ii^—Mn—N1—C11	78.9 (2)	Mn—N2—C5—C7	−1.1 (2)
N1^ii^—Mn—N1—C7	90.94 (16)	C1—N2—C5—C4	−0.3 (3)
N1^ii^—Mn—N1—C11	−91.8 (2)	C1—N2—C5—C7	179.10 (19)
N2^ii^—Mn—N1—C7	163.01 (16)	N2—C1—C2—C3	0.4 (4)
N2^ii^—Mn—N1—C11	−19.7 (2)	N2—C1—C2—C6	−179.4 (3)
Cl1—Mn—N2—C1	1.11 (17)	C1—C2—C3—C4	−0.7 (4)
Cl1—Mn—N2—C5	−178.73 (15)	C6—C2—C3—C4	179.1 (3)
N1—Mn—N2—C1	−179.78 (19)	C2—C3—C4—C5	0.5 (4)
N1—Mn—N2—C5	0.38 (15)	C3—C4—C5—N2	0.0 (4)
Cl1^ii^—Mn—N2—C1	−92.95 (17)	C3—C4—C5—C7	−179.4 (2)
Cl1^ii^—Mn—N2—C5	87.21 (16)	N2—C5—C7—N1	1.4 (3)
N1^ii^—Mn—N2—C1	90.71 (17)	N2—C5—C7—C8	−176.9 (2)
N1^ii^—Mn—N2—C5	−89.13 (16)	C4—C5—C7—N1	−179.2 (2)
N2^ii^—Mn—N2—C1	134.05 (18)	C4—C5—C7—C8	2.5 (3)
N2^ii^—Mn—N2—C5	−45.8 (3)	N1—C7—C8—C9	1.5 (3)
Mn—N1—C7—C5	−1.1 (2)	C5—C7—C8—C9	179.7 (2)
Mn—N1—C7—C8	177.24 (16)	C7—C8—C9—C10	−1.8 (4)
C11—N1—C7—C5	−178.5 (2)	C8—C9—C10—C11	0.8 (4)
C11—N1—C7—C8	−0.2 (3)	C8—C9—C10—C12	−178.3 (3)
Mn—N1—C11—C10	−178.1 (2)	C9—C10—C11—N1	0.6 (4)
C7—N1—C11—C10	−0.9 (4)	C12—C10—C11—N1	179.7 (3)

Symmetry codes: (i) −*x*+1, *y*, −*z*+3/2; (ii) −*x*+1, *y*, −*z*+1/2; (iii) −*x*+1, −*y*+1, −*z*+1; (iv) −*x*+3/2, −*y*+1/2, −*z*+1; (v) *x*−1/2, −*y*+1/2, *z*−1/2.

### Hydrogen-bond geometry (Å, °)

**Table:**

*D*—H···*A*	*D*—H	H···*A*	*D*···*A*	*D*—H···*A*
O1—H1A···Cl1	0.84	2.42	3.243 (3)	168
O1—H1B···Cl1^iii^	0.81	2.73	3.358 (4)	136
O2—H2A···O1	0.86	2.16	2.951 (6)	153
C3—H3···O1^v^	0.93	2.49	3.257 (5)	140
C6—H6A···Cl1^iii^	0.96	2.79	3.717 (4)	162

Symmetry codes: (iii) −*x*+1, −*y*+1, −*z*+1; (v) *x*−1/2, −*y*+1/2, *z*−1/2.
